# How we do it: robotic-assisted distal ureterectomy with ureteral reimplantation

**DOI:** 10.1590/S1677-5538.IBJU.2021.0207

**Published:** 2021-06-25

**Authors:** Christopher Pulford, Kevin Keating, Matthew Rohloff, David Peifer, Richard Eames, Thomas Maatman

**Affiliations:** 1 Midwestern University Arizona College of Osteopathic Medicine AZ USA Arizona College of Osteopathic Medicine, Midwestern University, AZ, USA.; 2 University of Michigan Department of Urology MI USA Department of Urology, Metro Health, University of Michigan, MI, USA.

## Abstract

**Background:**

High risk upper tract urothelial carcinoma (UTUC) is typically managed with radical nephroureterectomy, however, renal preservation can be attempted when UTUC is localized to the distal ureter in the presence of chronic kidney disease (1–3). Distal ureterectomy is typically managed with a ureteral reimplantation and psoas hitch in order to maintain urothelial continuity, to avoid comprising the contralateral ureter, and reducing risk of chronic urinary tract infections and electrolyte abnormalities (4). We present our case of distal ureteral UTUC managed robotically with a distal ureterectomy with ureteral reimplantation.

**Technique and Follow-Up:**

Initially, an Orandi needle on a resectoscope circumscribed the left ureteral orifice. Next, robotically, the retroperitoneum was exposed and a left sided pelvic lymphadenectomy was completed. The left ureter was mobilized and the diseased ureteral segment was transected. The mobilized bladder was sutured to psoas fascia. After a cystotomy, the ureter was re-anastomosed to the bladder. The patient was discharged on postoperative day three and re-evaluated one week later with a cystogram. Final pathology was downgraded to non-invasive low-grade papillary urothelial carcinoma with negative lymph nodes and margins.

**Conclusion:**

High risk UTUC localized to the distal ureter in the setting of chronic kidney disease can be managed with a distal ureterectomy (3). Robotic distal ureterectomy with ureteral reimplantation can be assisted by an Orandi needle to achieve negative margins. Utilizing a robotic technique can offer challenges with the ureteral spatulation and reanastomosis (5–7). By fixating the ureter to the bladder prior to reanastomosis, our technique offers a solution for these difficulties.

## SOURCE OF WORK

All work is original from TM and Metro Health - University of Michigan.

Authors have received and archived patient consent for video recording/publication in advance of video recording procedure.
